# Diverse HCV Strains And HIV URFS Identified Amongst People Who Inject Drugs In India

**DOI:** 10.1038/s41598-020-64309-5

**Published:** 2020-04-29

**Authors:** Mary A. Rodgers, Selvamurthi Gomathi, Ana Vallari, Shanmugam Saravanan, Gregory M. Lucas, Shruti Mehta, Sunil S. Solomon, Gavin A. Cloherty

**Affiliations:** 1 Abbott Diagnostics, Infectious Disease Research, Abbott Park, USA; 2 YR Gaitonde Centre for AIDS Research and Education, Chennai, India; 3 Johns Hopkins University School of Medicine, Baltimore, USA; 4 Johns Hopkins Bloomberg School of Public Health, Baltimore, USA

**Keywords:** Virology, Medical research, Epidemiology

## Abstract

Although the prevalences of HIV and HCV are significantly higher amongst PWID in India compared to the general population, the strains circulating within this group have not been well-characterized. Through subgenomic sequencing of viruses present in residual plasma from an HIV/HCV prevalence study conducted amongst PWID across five cities in India in 2016–2017, a total of N = 498 HCV and N = 755 HIV strains were classified from N = 975 study participants. Considerable HCV diversity was identified, with different strains predominating in each region of the country. Overall, the most common strain was genotype 3a (39.0%), with genotypes 1a (26.9%), 1b (3.0%), 1c (0.2%), 3b (20.7%), 3i (2.0%), 4a (0.2%), 4d (1.0%), 6 (1.8%), 6n (4.8%), 6 v (0.2%) and one unclassifiable recombinant specimen (0.2%) also identified. The majority of the HIV specimens were subtype C (96.7%), although subtype A (0.4%), CRF01_AE (0.4%) and unique recombinant forms (URFs, 2.5%) were also detected. Notably, the geographical restriction of HIV subtype A and CRF01_AE, and HCV genotypes 4 and 6 to specific sites suggests distinct novel introductions of HIV and HCV into PWID populations, potentially via drug trafficking routes from neighboring countries where these strains are common.

## Introduction

The global hepatitis C virus (HCV) and human immunodeficiency virus (HIV) pandemics affect 71 and 36.9 million people, respectively, who are chronically infected worldwide^1,2^. Chronic HCV infection damages the liver over the course of decades, putting HCV patients at higher risk for developing cirrhosis and hepatocellular carcinoma^3^. The clinical consequences of HCV infection are mitigated by treatment with direct acting antivirals (DAAs), which have cured more than 3 million people since 2015^4^. Therefore, screening to identify positive patients in need of treatment is a critical first step towards HCV elimination. Likewise, the suppression of HIV achieved by antiretroviral therapy (ART) reduces the impact of HIV infection on CD4 T cell counts and improves life expectancy^2^. Once on ART, HIV patients are less likely to transmit the virus, making HIV diagnostic testing and treatment the central pillars of HIV control efforts^5^.

The incredible sequence diversity of HIV and HCV presents unique challenges to diagnostic tests, which fundamentally rely on sequence conservation to deliver accurate results. With 2 types, 4 HIV-1 groups, 9 Group M subtypes, and over 100 recognized circulating recombinant forms (CRFs), HIV is a highly divergent virus^6^. Part of this diversity is driven by recombination between strains in co-infected individuals, which has led to the establishment of CRFs and unique recombinant forms (URFs) as increasingly prevalent strains globally^6^. Although HCV is also capable of recombination, it is far less common with mostly isolated cases of recombinant strains identified and only one recognized circulating recombinant form; CRF01_1b2k^7–9^. However, HCV strains are more divergent than HIV strains^10^, with 8 major genotypes and more than 80 subgenotypes identified to date^11–14^. Global molecular surveillance of circulating HIV and HCV strains is essential for defining the true extent of viral diversity and to ensure that diagnostic tests, vaccines, and therapeutics keep pace with viral evolution.

Considerable viral diversity exists in India, where the newly recognized HCV genotype 8 is expected to be endemic after its discovery amongst Indian immigrants to Canada from the Punjab region^12^. Previous HCV diversity studies have reported that nearly all HCV genotypes are present in India, with genotypes 1 and 3 being the most common, and genotypes 2, 4 and 6 found less frequently^15–19^. While recombination is rare for HCV, a 3a/1a recombinant strain has been identified in Kolkata amongst PWID, suggesting that other recombinant strains may also be circulating in India^20^. In contrast to HCV diversity studies, previous HIV diversity studies in India have been small in scale (13–130 participants), limited to a single subgenomic region, and largely focused on a single city or region^21–28^. The predominant strain of HIV in all regions of India is subtype C^21–28^. However, URFs are frequent (32.73%) and subtype B has also been found (3.34%) in the Northeastern region near the ‘golden triangle’ of opium production^29^. Subtypes A, B, and B/C recombinants have also been identified in the North^25,27,28^.

The alarmingly high prevalences of HIV and HCV amongst PWID in India, which exceed 60% in some cities, indicate that additional attention must be paid to these groups to end the local HIV and HCV epidemics^30,31^. To address this gap in knowledge, the following study presents the first large scale characterization of HIV and HCV viral strains circulating amongst PWID in five cities across Northern, Northeastern, and Central India.

## Materials and Methods

### Study population

Blood samples and surveys were collected from PWID (1000/site) recruited via respondent driven sampling in 2016–2017 as previously described^31^. The study was approved by the institutional review boards (IRB) of YRGCARE in Chennai, India, and Johns Hopkins University School of Medicine in Baltimore. The study survey participants provided verbal informed consent as approved by the IRBs. Individuals were eligible to participate if they were 18 years or older. All methods were carried out in accordance with the protocols approved by the IRB. A subset of samples with sufficient viral load (>3.5 log IU/ml) and remaining volume from 5 cities were included in this analysis: Amritsar and Delhi in North India, Kanpur in Central India and Imphal and Aizawl in Northeastern India. Samples were tested for HIV on-site using three rapid tests as previously described^31^ as part of the assessment of a cluster randomized trial and results were provided to participants with appropriate pre- and post-test counseling^32^. HIV RNA quantification was performed on all samples testing positive for HIV using the RealTi*m*e HIV-1 viral load assay (Abbott Molecular Diagnostics, Des Plaines, IL, USA). Stored specimens were tested for antibodies to HCV and samples testing positive for HCV antibodies were tested for HCV RNA using the RealTi*m*e HCV viral load assay (Abbott Molecular Diagnostics, Des Plaines, IL, USA).

### Sanger sequencing

RNA was extracted from plasma specimens on the m2000sp instrument (Abbott Molecular Diagnostics, Des Plaines, IL, USA). A 676 nucleotide region of the HIV *env* immunodominant region (IDR), a 1009 nucleotide region of the HIV *pol* integrase (IN), and/or a 750 nucleotide region of the HCV 5′UTR-core region was amplified by RT-PCR for sequencing by Sanger methods as previously described^33,34^. The Genbank accession numbers for the *pol* IN sequences are MN697000-MN697738, the *env* IDR sequences are MN378645-MN379312, and the HCV 5UTR-*core* sequences are MN697739-MN698236.

### HCV Phylogenetic classification

**Figure 1:**
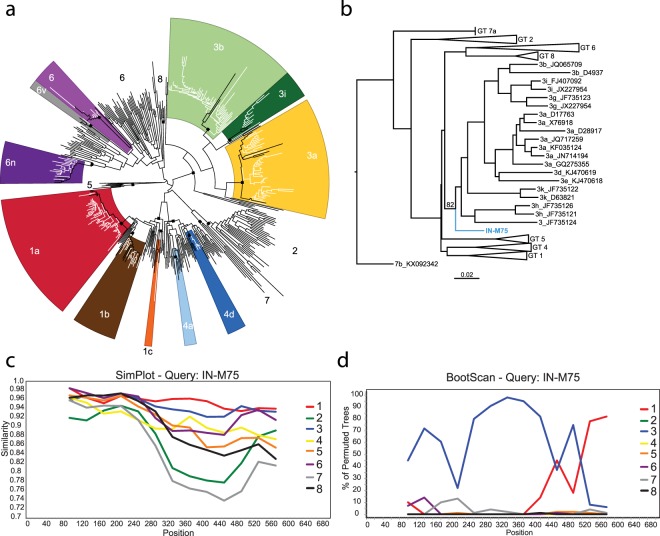
HCV phylogenetic and recombinant analysis. (**a**) A representative subset of N = 212 study 5′UTR-core region sequences are shown as white branches on a Neighbor-Joining tree. Reference genotypes for each clade are shown on the outer ring of the radial tree and branches with study sequences in them are highlighted in color. Relevant nodes with bootstrap >70 are labeled with a black square. (**b**) The recombinant sequence (IN-M75) is shown in blue with reference strains in black. The classifications and Genbank accession numbers of related strains are labeled. A similarity plot (**c**) and a bootscan plot (**d**) for the recombinant sequence using a 200 basepair window, 40 basepair steps, Kimura 2-paramter, and T/t 2.0 conditions. Consensus sequences for reference strains were used for these plots inclusive of all subgenotypes for each classification.

Groups of 50–200 sequences were aligned to reference strains for genotypes 1–8 (accession numbers listed in Supplemental Table 1) by MUSCLE in Sequencher v5.4.6 (Gene Codes, Ann Arbor, MI). Alignments were degapped and trimmed to the longest query sequence length in Bioedit v7.2.5^35^. Neighbor-joining phylogenetic trees were prepared using Phylip v3.5 as previously described^34^. Classifications were assigned to closest references with a bootstrap of >70. To improve visualization in Fig. 1, a subset of N = 212 study sequences were included that encompassed the full diversity of all HCV samples in the study. Phylogenetic tree figures were prepared using FigTree 1.4.2 and Adobe Illustrator CC 2018.

**Table 1:** Table of HCV classifications identified.

HCV genotype	N	%
1a	134	26.91
1b	15	3.01
1c	1	0.20
3a	194	38.96
3b	103	20.68
3i	10	2.01
4a	1	0.20
4d	5	1.00
6	9	1.81
6n	24	4.82
6 v	1	0.20
U	1	0.20
Total	498

### HIV Phylogenetic classification

**Figure 2:**
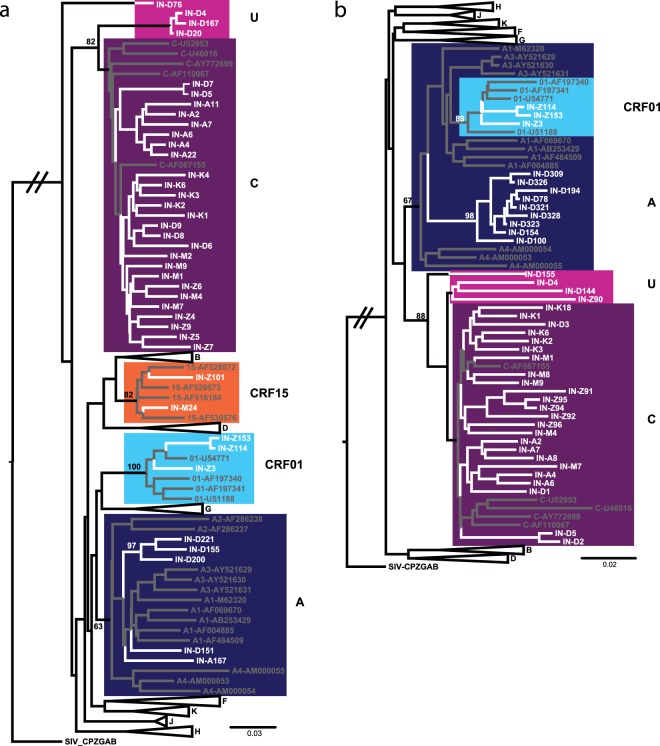
HIV phylogenetic analysis. Neighbor-Joining trees are shown for study sequences representative of the full HIV diversity identified in the *pol* IN (**a**) (N = 40) and *env* IDR (**b**) regions (N = 40). Study sequences branches are white and reference strains are gray or black with classifications and Genbank accession numbers included in branch labels. Clades containing study sequences are highlighted with color boxes and relevant nodes are labeled with bootstrap values.

Individual sequences were aligned to reference strains for HIV-1 Group M strains A-K, and CRF01–96 by MAFFT. Alignments were degapped and trimmed to the query sequence length. Neighbor-joining phylogenetic trees and classifications were assigned as described above for HCV. The reference and sample sequence list in the alignments used to generate trees shown in Fig. 2 was reduced to improve visualization while representing the full range of viral diversity encountered in study specimens. In particular, CRF branches that did not include study sequences were removed. Phylogenetic tree figures were prepared using FigTree 1.4.2 and Adobe Illustrator CC 2018.

### Recombinant analysis

For HCV and HIV sequences branching basal to references or with bootstrap values <70, recombinant analysis was performed using Simplot version 3.5.1 software to identify breakpoints or unclassifiable regions.

### Serological testing

To compare serological assay performance, all HCV and HIV specimens with sufficient remaining volume underwent additional serological testing on the ARCHITECT i2000 instrument (Abbott Laboratories, Abbott Park, IL, USA). HCV specimens were screened with the anti-HCV and HCV antigen tests and HIV specimens were screened with the HIV Ag/Ab Combo test according to the package inserts. Retesting of initial reactives and confirmatory testing recommended in the package inserts was not done.

## Results

In an HIV/HCV study conducted amongst PWID in India, plasma specimens were collected from N = 5000 participants across five cities as part of the evaluation assessment of a cluster randomized trial: Amritsar, Aizawl, Delhi, Imphal, and Kanpur (1000/site)^32^. Leftover specimens with sufficient volume were selected for further molecular characterization in this study if viral load was at least 3 log 10 IU/ml (HCV) or 3 log 10 copies/ml (HIV). In total, N = 975 leftover plasma specimens with sufficient volume and viral load for sequencing that were collected from N = 477 HIV antibody positive, N = 220 HCV antibody positive, and N = 278 dual positive study participants were sequenced to identify the viral strains present (Supplemental Fig. 1). HIV viral loads for these specimens ranged from 3.5 to 6.9 log_10_ copies/ml (median 4.8 log_10_ copies/ml) and HCV viral loads for included specimens ranged from 3.8 to 7.3 log_10_ IU/ml (median 5.6 log_10_ IU/ml).

**Figure 3:**
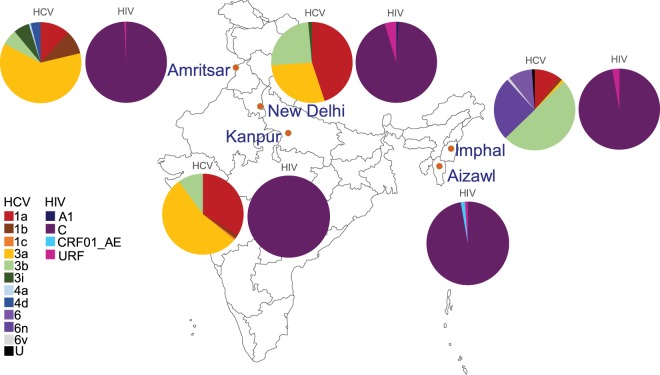
Pie charts indicating the relative prevalence of each indicated virus classification are shown adjacent to the study site where they were identified. The map of India was obtained from https://d-maps.com/carte.php?num_car=4183&lang=en.

Classification of the HCV 5′UTR-core regions of N = 498 specimens identified a diverse set of strains circulating in Indian PWID (Fig. 1, Table 1). Overall, the most common genotype was 3a (N = 194, 39%), followed by 1a (N = 134, 27%), with genotypes 1b, 1c, 3b, 3i, 4a, 4d, 6, 6n, 6 v, and one unclassifiable (U) sequence also present (Fig. 1, Table 1). A comparison of the genotype distributions in each city revealed that unique strains predominated in different regions of India (Fig. 3). In the Northeastern city of Imphal, genotypes 3b (50%) and 6n (25%) were most common (Fig. 3). Notably, Imphal was the only site where genotype 6 strains were found in this study. An unclassifiable strain was also identified from Imphal that branched basal to all genotype 3 references and displayed evidence of recombination with genotype 1 (Fig. 1), although this strain was not related to the 3a/1a recombinant previously identified amongst PWID in Kolkata^20^. In contrast, the predominant strains in the Northern city of Amritsar were 3a (61%) and 1a (12%) (Fig. 3). Additional diversity was also observed in Amritsar, with genotype 4 strains exclusively found at this site (Fig. 3). Although New Delhi and Kanpur are both located in Central India, the most highly prevalent strains of HCV were 3a (54%) in Kanpur and 1a (45%) in New Delhi, indicating that city-level differences exist within the Central region (Fig. 3). This is further supported by the detection of genotypes 1b and 1c in Kanpur but not in New Delhi (Fig. 3).

Unlike the diversity encountered within the HCV sequences, the HIV sequences sampled from these same cities were substantially less diverse, although recombinant strains were identified. Either the HIV *env* IDR (N = 16 samples), *pol* IN (N = 87 samples), or both regions (N = 652 samples) were sequenced from a total of N = 755 HIV positive specimens. Altogether, N = 668 *env* IDR and N = 739 *pol* IN sequences were classified, the majority of which were classified as HIV subtype C (728, 96.7%) (Fig. 2, Table 2). A small number of URFs were identified with discordant IDR and IN classifications or recombination detected in at least one region (19, 2.5%). The majority of the URFs were A/C recombinants (13, 68.4%), with B/C or CRF15 recombinants (3, 15.8%) and unclassifiable regions also identified (3, 15.8%) (Table 3). URFs containing at least one region classified as a CRF were only identified in the Northeastern city of Aizawl (CRF15, CRF01) (Table 3). Overall, URFs were identified in all of the sites except Kanpur, with the highest prevalence found in New Delhi (15, 4.7%) (Fig. 3). Three CRF01_AE infections were identified exclusively in Aizawl (0.4%), and New Delhi was the only site were subtype A infections were found (N = 3, 0.4%), consistent with unique localized epidemics (Fig. 3).

**Table 2:** Table of HIV classifications identified.

HIV subtype	N	%
A	3	0.40
C	730	96.69
CRF01	3	0.40
URF	19	2.52
Total	755

**Table 3:** HIV recombinants.

Specimen	Site^+^	HCV GT	Overall HIV	*env* IDR classification	*pol* IN classification
IN-D200	DH		URF	A	C
IN-M24	IM	3b	URF	CRF15	C
IN-Z90	AZ		URF	C	URF_BC
IN-D194	DH	3b	URF	C	A
IN-A167	AM	3a	URF	A	C
IN-D144	DH	3a	URF	C	URF_AC
IN-D321	DH		URF	C	A
IN-D78	DH		URF	C	A
IN-D154	DH		URF	C	A
IN-D309	DH		URF	C	A
IN-D151	DH		URF	A	C
IN-D221	DH		URF	A	C
IN-D167	DH		URF	URF_A1C	C
IN-D155	DH		URF	A	URF_A1C
IN-D100	DH		URF	C	A
IN-D76	DH	1a	URF	U	C
IN-Z101	AZ		URF	CRF15	C
IN-D4	DH		URF	URF_CU	URF_CU
IN-D20	DH	1a	URF	URF_CU	C

+New Delhi (DH), Imphal (IM), Aizawl (AZ), Amritsar (AM).

The sequences generated from the N = 278 PWID that were co-infected with HIV and HCV provided an opportunity to directly compare viral diversity for both viruses within the same individuals. The same genotypes and subtypes found in the overall study (Table 2) were also represented within the co-infected group; namely, HCV genotypes 1, 3, 4, 6 and HIV subtypes A, C, URF (Table 4). Remarkably, the prevalences of each HIV and HCV strain within the co-infected subset were nearly identical to the overall proportions of each classification in the complete sample (Table 4), suggesting that infection with one virus (HIV or HCV) did not influence the strain acquired in a second viral co-infection.

**Table 4:** Co-infection classifications (N).

		HIV			
		A	C	URF	Total
HCV	1a		88	2	90
	1b		8		8
	3a	2	106	2	110
	3b		55	2	57
	3i		7		7
	4a		1		1
	4d		2		2
	6		1		1
	6n		2		2
	Total	2	270	6	278

**Figure 4:**
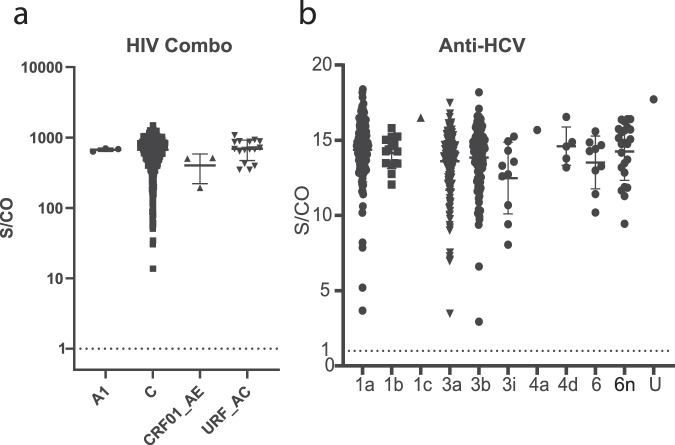
Signal to cutoff (S/CO) values are plotted for study samples tested with HIV Combo (**a**) and Anti-HCV (**b**) assays. The mean result for each virus classification is represented by a horizontal bar and error bars indicate standard deviation from the mean. A dashed line marks the positive cutoff for each test.

All characterized specimens with residual volume were screened with additional serological assays to evaluate their performance with diverse clinical specimens from India. Amongst the N = 674 HIV specimens available for testing on the ARCHITECT HIV Combo assay, all specimens were reactive, giving an assay sensitivity of 100% (Fig. 4). Likewise, the sensitivity of the ARCHITECT Anti-HCV assay was 100%, with all N = 488 HCV specimen with sufficient volume detected as reactive (Fig. 4). A total of N = 474 remaining HCV specimens were subsequently screened with the ARCHITECT HCV antigen assay and the results were compared to HCV viral load. A total of N = 470 samples were detected as reactive or grayzone-reactive (between 0.80 to 0.99 S/CO), resulting in an assay sensitivity of 99.16%, consistent with previous reports^36–42^. Unfortunately, replicate testing could not be completed for the four samples that were nonreactive due to sample depletion. The sequences for these four samples confirmed that they were of the most common genotypes found in the study; 1a, 3a, and two 3b samples, suggesting that genotype alone could not explain the nonreactive results for these samples. Furthermore, point mutations in the detection epitopes of these specimens were also present in samples of similar viral load with detectable HCV antigen.

To characterize the prevalence of HIV drug resistance mutations circulating in PWID in India, the N = 742 HIV *pol* IN sequences were further examined for the presence of major integrase inhibitor (INSTI) resistance mutations as defined by the Stanford HIV drug resistance database (HIVDB)^43^. The overall prevalence of INSTI resistance mutations was low, with N = 4 (0.5%) specimens identified with a pure INSTI mutation (E92Q, E138A, or R263K) and N = 7 (0.9%) with a mixture of wildtype and resistance mutations at the amino acid 92 position. Since INSTIs were not used by public-sector clinics during the period of this study^44^, the identified resistance mutations are likely due to the natural level of variation in the *pol* IN gene, although it is also possible that these strains were imported. The detection of these INSTI resistance mutations at three different sites (Aizawl, Delhi, and Amritsar) confirms that INSTI resistance was not localized to a single city.

## Discussion

This is the first large scale viral diversity study conducted amongst PWID in India, which generated N = 1905 viral sequences from N = 975 PWID participants located in five cities across the Northern, Central, and Northeastern regions of India. The diverse HIV and HCV strains identified in this study are consistent with those identified in other cities and study cohorts in India^15–19,21–24,26–29^. Given that the prevalence of HIV and HCV are considerably higher in PWID (2.4–64.9%)^30^ than in non-PWID populations in India (0.22–0.88%)^45,46^, it remains possible that the strains in the PWID group are a reservoir for the epidemic in the general population in India. Our data can inform treatment and prevention strategies targeted towards PWID to enable the greatest impact on the local HIV and HCV epidemics in India. In particular, the low prevalence of INSTI resistance mutations observed in this study suggests that INSTI-based regimens could be successful in India as a first-line HIV treatment option. With DAA cures available for HCV, treatment is an important component of the HCV elimination goal set by the World Health Organization (WHO)^47^. As part of this goal, 90% of all HCV infections should be diagnosed by 2030, with 80% of eligible patients receiving treatment^47^. Sequence diversity has the potential to impact both of these elimination activities; diversity can challenge the accuracy of diagnostic tests^48,49^ and the efficacy of some DAA combinations^50,51^. Notably, pan-genotypic DAA combinations have the strongest sustained virological response (SVR) rates for genotype 3^51,52^, which was the most common genotype in our study (Table 1).

Although considerable HCV diversity and HIV URFs were identified in our study, this is likely an underestimation of the true genetic diversity for each virus because subgenomic sequences cannot capture the full extent of recombination in a given sample^53^. Therefore, we propose that complete genome sequencing should be conducted to identify the true prevalence of viral recombinants in India. Since recombination requires co-infection with multiple strains, we predict that the higher rates of HIV and HCV incidence amongst PWID would result in higher rates of novel recombination events in this population. Given that the overwhelming majority of the HIV strains were all subtype C, the possibility remains that recombination events between such similar strains could be difficult to detect. However, the identification of at least one recombinant HCV strain by subgenomic sequencing suggests that other recombinants could likely be identified in whole genome characterization studies.

Geographical stratification of the HIV and HCV strains identified herein confirmed that higher levels of viral diversity were present in cities near borders with neighboring countries where drug trafficking routes exist^54^. Notably, the HCV strains that predominated in the Northeastern city of Imphal varied dramatically from those identified in the Northern border city of Amritsar (Fig. 3). Furthermore, a greater number of different HCV strains were encountered in both of these cities (7–8 total) than were found in either of the Central cities of Kanpur (5) or New Delhi (4) (Fig. 3). These results support the hypothesis that new strains have been imported to India via the Golden Triangle and Golden Crescent opium trade routes^55^. Indeed, the non-C HIV classifications identified in our study are commonly found in Southeast Asia (CRF01_AE), East Asia (subtype B and URFs), and Pakistan (subtype A)^6,56^ (Table 2). Although HCV samples were not available for sequencing from the Aizawl site in Northeastern India, this was the only location where CRF01_AE HIV strains were identified, which is consistent with the high prevalence of CRF01_AE in Southeast Asia^6^. Likewise, the HCV genotypes 4 and 6 strains identified in the North and Northeastern sites in India are more prevalent in East Asia^57^.

An important purpose of this study was to characterize viral diversity in India towards the ultimate goal of using diverse viral strains in circulation as a challenge for diagnostic tests. By pairing HIV and HCV sequence data with serological and viral load data, this surveillance study demonstrated that the ARCHITECT HIV Combo, Anti-HCV, and HCV antigen tests were able to detect a diverse range of HCV genotypes and HIV strains present in PWID in India. In addition to reaffirming the sensitivities of these assays in a unique population, these data also highlight the importance of continued vigilance against the threats posed by viral evolution to the accuracy of diagnostic tests. Given that diagnostic screening for HIV and HCV is the first step in treatment and prevention efforts, a sustained effort to track viral diversity remains a critical component of global strategies to end these pandemics.

## Supplementary information

Supplementary information.

## Data Availability

All sequences in this study have been deposited in Genbank. Genbank accession numbers for the *pol* IN sequences are MN697000-MN697738, the *env* IDR sequences are MN378645-MN379312, and the HCV 5UTR-*core* sequences are MN697739-MN698236.
