# Supplementary material for: Diverse HCV Strains And HIV URFS Identified Amongst People Who Inject Drugs In India
Source: Sci Rep. 2020 Apr 29;10:7214. doi: 10.1038/s41598-020-64309-5 (PMC7190742; doi:10.1038/s41598-020-64309-5)
Supplement: Supplementary file 1 — Supplementary information. [file 41598_2020_64309_MOESM1_ESM.docx]

**DIVERSE HCV STRAINS AND HIV URFS IDENTIFIED AMONGST PEOPLE WHO INJECT DRUGS IN INDIA**

Mary A Rodgers^1*^, Selvamurthi Gomathi^2^, Ana Vallari^1^, Shanmugam Saravanan ^2^, Gregory M Lucas^3^, Shruti Mehta^4^, Sunil S Solomon^2,3,4^, Gavin A Cloherty^1^

1 Abbott Diagnostics, Infectious Disease Research, Abbott Park, USA

2 YR Gaitonde Centre for AIDS Research and Education, Chennai, India

3 Johns Hopkins University School of Medicine, Baltimore, USA

4 Johns Hopkins Bloomberg School of Public Health, Baltimore, USA

*Corresponding author: 100 Abbott Park Rd, Abbott Park, IL 60064, mary.rodgers@abbott.com

**SUPPLEMENTAL MATERIAL**

Supplemental Table 1. List of HCV reference sequence accession numbers

| Accession number | Classification |
| --- | --- |
| EF407457 | 1a |
| EU781749 | 1a |
| HQ850279 | 1a |
| AJ851228 | 1 |
| M62321 | 1a |
| M67463 | 1a |
| NC004102 | 1a |
| D90208 | 1b |
| EU781827 | 1b |
| EU781828 | 1b |
| M58335 | 1b |
| AY051292 | 1c |
| D14853 | 1c |
| KJ439768 | 1d |
| KC248194 | 1e |
| KJ439769 | 1e |
| AM910652 | 1g |
| KJ439770 | 1g |
| KC248198 | 1h |
| KC248199 | 1h |
| KJ439772 | 1i |
| KJ439773 | 1j |
| KC248195 | 1 |
| KJ439776 | 1 |
| KJ439777 | 1 |
| KJ439779 | 1 |
| KJ439780 | 1 |
| KJ439774 | 1k |
| KC248193 | 1l |
| KC248196 | 1l |
| KC248197 | 1l |
| KJ439778 | 1m |
| KJ439782 | 1m |
| KJ439775 | 1n |
| KJ439781 | 1n |
| AB047639 | 2a |
| D00944 | 2a |
| HQ639944 | 2a |
| AB030907 | 2b |
| AB661382 | 2b |
| D10988 | 2b |
| D50409 | 2c |
| JX227949 | 2c |
| JF735114 | 2d |
| JF735120 | 2e |
| KC844042 | 2f |
| KC844050 | 2f |
| DQ155561 | 2i |
| KC197229 | 2i |
| KC197230 | 2i |
| JF735110 | 2 |
| JF735116 | 2 |
| JF735117 | 2 |
| JF735118 | 2 |
| JF735119 | 2 |
| HM777358 | 2j |
| HM777359 | 2j |
| JF735113 | 2j |
| AB031663 | 2k |
| KC197236 | 2 |
| KC197237 | 2 |
| KC197239 | 2 |
| JX227953 | 2k |
| JF735111 | 2m |
| JX227967 | 2m |
| FN666428 | 2q |
| FN666429 | 2q |
| JF735115 | 2r |
| KC197238 | 2t |
| JF735112 | 2u |
| D28917 | 3a |
| GQ275355 | 3a |
| JN714194 | 3a |
| JQ717259 | 3a |
| D17763 | 3a |
| D49374 | 3b |
| JQ065709 | 3b |
| KJ470619 | 3d |
| KJ470618 | 3e |
| JF735123 | 3g |
| JX227954 | 3g |
| JF735121 | 3h |
| JF735126 | 3h |
| FJ407092 | 3i |
| JX227955 | 3i |
| JF735124 | 3J |
| D63821 | 3k |
| JF735122 | 3k |
| DQ418789 | 4a |
| DQ988074 | 4a |
| Y11604 | 4a |
| FJ462435 | 4b |
| FJ462436 | 4c |
| DQ418786 | 4d |
| EU392172 | 4d |
| FJ462437 | 4d |
| EF589161 | 4f |
| EU392174 | 4f |
| EU392175 | 4f |
| FJ025854 | 4 |
| FJ462432 | 4g |
| JX227971 | 4g |
| JF735127 | 4 |
| JF735129 | 4 |
| JF735130 | 4 |
| JF735131 | 4 |
| JF735132 | 4 |
| JF735134 | 4 |
| JF735135 | 4 |
| JF735138 | 4 |
| JX227964 | 4 |
| EU392171 | 4k |
| EU392173 | 4k |
| FJ462438 | 4k |
| FJ839870 | 4l |
| JX227957 | 4l |
| FJ462433 | 4m |
| JX227972 | 4m |
| FJ462441 | 4n |
| JX227970 | 4n |
| FJ462440 | 4o |
| JX227977 | 4o |
| FJ462431 | 4p |
| FJ462434 | 4q |
| FJ462439 | 4r |
| JX227976 | 4r |
| JF735136 | 4s |
| FJ839869 | 4t |
| HQ537009 | 4v |
| JX227960 | 4v |
| FJ025855 | 4w |
| FJ025856 | 4w |
| AF064490 | 5a |
| KC767832 | 5a |
| KC844046 | 5a |
| KF373567 | 5a |
| KJ925149 | 5a |
| Y13184 | 5a |
| KT595242 | 5 |
| EU246930 | 6a |
| HQ639936 | 6a |
| Y12083 | 6a |
| D84262 | 6b |
| EF424629 | 6c |
| KM504124 | 6c |
| D84263 | 6d |
| DQ278891 | 6 |
| DQ278893 | 6 |
| DQ314805 | 6e |
| EU246932 | 6e |
| DQ835760 | 6f |
| EU246936 | 6f |
| D63822 | 6g |
| DQ314806 | 6g |
| D84265 | 6h |
| KM252783 | 6h |
| KM504122 | 6h |
| DQ835762 | 6i |
| DQ835770 | 6i |
| DQ835761 | 6j |
| DQ835769 | 6j |
| JX183549 | 6 |
| JX183550 | 6 |
| JX183551 | 6 |
| JX183553 | 6 |
| JX183554 | 6 |
| JX183558 | 6 |
| KC844039 | 6 |
| KC844040 | 6 |
| D84264 | 6k |
| KJ470620 | 6 |
| KJ470621 | 6 |
| KJ470622 | 6 |
| KJ470623 | 6 |
| KJ470624 | 6 |
| KJ470625 | 6 |
| KJ567644 | 6 |
| KJ567648 | 6 |
| KJ567649 | 6 |
| KJ567650 | 6 |
| KJ567652 | 6 |
| EF424628 | 6l |
| JX183556 | 6l |
| DQ835766 | 6m |
| DQ835767 | 6m |
| DQ835768 | 6n |
| EU246938 | 6n |
| KC191671 | 6n |
| EF424627 | 6o |
| EU246934 | 6o |
| EF424626 | 6p |
| KM252785 | 6p |
| EF424625 | 6q |
| KM504115 | 6q |
| KM504116 | 6q |
| EU408328 | 6r |
| KM252786 | 6r |
| EU408329 | 6s |
| KM252787 | 6s |
| EF632071 | 6t |
| EU246939 | 6t |
| EU246940 | 6u |
| KM252788 | 6u |
| EU158186 | 6v |
| EU798760 | 6v |
| EU798761 | 6v |
| DQ278892 | 6w |
| EU643834 | 6w |
| EU643836 | 6w |
| E408330 | 6xa |
| E408331 | 6xa |
| E408332 | 6xa |
| J183552 | 6xb |
| K567645 | 6xb |
| K567651 | 6xc |
| K252789 | 6xd |
| K252790 | 6xd |
| K252791 | 6xd |
| J183557 | 6xe |
| K252792 | 6xe |
| K567646 | 6xf |
| K567647 | 6xf |
| E108306 | 7a |
| KX092342 | 7b |
| KU861171 | 7 |
| KX172138 | 7 |
| MH940291 | 7 |
| MH590698 | 8 |
| MH590699 | 8 |
| MH590700 | 8 |
| MH590701 | 8 |

Supplemental Table 2. List of HIV reference sequence accession numbers

| Accession number | Classification |
| --- | --- |
| X52154 | SIV |
| AF197340 | CRF01 |
| U51188 | CRF01 |
| AF197341 | CRF01 |
| U54771 | CRF01 |
| AJ286133 | CRF02 |
| AF063224 | CRF02 |
| L39106 | CRF02 |
| AF107770 | CRF02 |
| AF414006 | CRF03 |
| AF193276 | CRF03 |
| AF193277 | CRF03 |
| AF119820 | CRF04 |
| AF119819 | CRF04 |
| AF049337 | CRF04 |
| AY227107 | CRF05 |
| AF193253 | CRF05 |
| AF076998 | CRF05 |
| AJ288982 | CRF06 |
| AJ245481 | CRF06 |
| AJ288981 | CRF06 |
| AF064699 | CRF06 |
| AF286226 | CRF07 |
| AF286230 | CRF07 |
| AX149647 | CRF07 |
| AF286229 | CRF08 |
| AY008715 | CRF08 |
| AY008716 | CRF08 |
| AY008717 | CRF08 |
| AY093603 | CRF09 |
| AY093604 | CRF09 |
| AY093605 | CRF09 |
| AY093607 | CRF09 |
| AF289548 | CRF10 |
| AF289549 | CRF10 |
| AF289550 | CRF10 |
| AF492624 | CRF11 |
| AF492623 | CRF11 |
| AJ291719 | CRF11 |
| AJ291720 | CRF11 |
| AJ291718 | CRF11 |
| AF179368 | CRF11 |
| AF408629 | CRF12 |
| AF408630 | CRF12 |
| AF385936 | CRF12 |
| AF385934 | CRF12 |
| AF385935 | CRF12 |
| AY371154 | CRF13 |
| AF460972 | CRF13 |
| AF460974 | CRF13 |
| AF423756 | CRF14 |
| AF423757 | CRF14 |
| AF423758 | CRF14 |
| AF423759 | CRF14 |
| AF450096 | CRF14 |
| AF450097 | CRF14 |
| AF516184 | CRF15 |
| AF529572 | CRF15 |
| AF529573 | CRF15 |
| AF530576 | CRF15 |
| AF286239 | CRF16 |
| AF457060 | CRF16 |
| AY945736 | CRF16 |
| AY037275 | CRF17 |
| AY037277 | CRF17 |
| AY037281 | CRF17 |
| EU581824 | CRF17 |
| EU581825 | CRF17 |
| AF377959 | CRF18 |
| AY586541 | CRF18 |
| AY894993 | CRF18 |
| AY586540 | CRF18 |
| AY588971 | CRF19 |
| AY588970 | CRF19 |
| AY894994 | CRF19 |
| AY586544 | CRF20 |
| AY586545 | CRF20 |
| DQ020274 | CRF20 |
| AY945737 | CRF21 |
| AF457051 | CRF21 |
| AF457072 | CRF21 |
| AY371159 | CRF22 |
| AY371165 | CRF22 |
| AY037284, AY037285 | CRF22 |
| EU743963 | CRF22 |
| GQ229529 | CRF22 |
| KP109500 | CRF22 |
| AY900571 | CRF23 |
| AY900572 | CRF23 |
| AY900575 | CRF24 |
| AY900574 | CRF24 |
| AY900576 | CRF24 |
| DQ826726 | CRF25 |
| AY371169 | CRF25 |
| EU693240 | CRF25 |
| EU697906 | CRF25 |
| EU697908 | CRF25 |
| FM877777 | CRF26 |
| FM877780 | CRF26 |
| FM877781 | CRF26 |
| FM877782 | CRF26 |
| AM851091 | CRF27 |
| AJ404325 | CRF27 |
| AM851090 | CRF27 |
| DQ085872 | CRF28 |
| DQ085873 | CRF28 |
| DQ085874 | CRF28 |
| AY455778 | CRF29 |
| AY771590 | CRF29 |
| DQ085871 | CRF29 |
| DQ085876 | CRF29 |
| AJ508597 | CRF30 |
| AY727526 | CRF31 |
| AY727527 | CRF31 |
| EF091932 | CRF31 |
| AY535659 | CRF32 |
| AY535660 | CRF32 |
| DQ167215 | CRF32 |
| DQ366659 | CRF33 |
| DQ366660 | CRF33 |
| DQ366661 | CRF33 |
| DQ366662 | CRF33 |
| EF165539 | CRF34 |
| EF165540 | CRF34 |
| EF165541 | CRF34 |
| EF158043 | CRF35 |
| EF158040 | CRF35 |
| EF158041 | CRF35 |
| EF158042 | CRF35 |
| AY371144 | CRF36 |
| EF087995 | CRF36 |
| EF087994 | CRF36 |
| AF377957 | CRF37 |
| KP718917 | CRF37 |
| EF116594 | CRF37 |
| FJ213783 | CRF38 |
| FJ213781 | CRF38 |
| FJ213782 | CRF38 |
| FJ213780 | CRF38 |
| EU735534 | CRF39 |
| EU735536 | CRF39 |
| EU735535 | CRF39 |
| EU735540 | CRF40 |
| EU735538 | CRF40 |
| EU735537 | CRF40 |
| EU735539 | CRF40 |
| KX907411 | CRF41 |
| KX907417 | CRF41 |
| KX907430 | CRF41 |
| EU170136 | CRF42 |
| EU170150 | CRF42 |
| EU170142 | CRF42 |
| EU170144 | CRF42 |
| EU697904 | CRF43 |
| EU697905 | CRF43 |
| EU697907 | CRF43 |
| EU697909 | CRF43 |
| AY536235 | CRF44 |
| FJ358521 | CRF44 |
| EU448295 | CRF45 |
| FN392874 | CRF45 |
| FN392875 | CRF45 |
| FN392876 | CRF45 |
| FN392877 | CRF45 |
| DQ358801 | CRF46 |
| HM026456 | CRF46 |
| HM026457 | CRF46 |
| FJ670529 | CRF47 |
| GQ372987 | CRF47 |
| KC473840 | CRF47 |
| KJ849798 | CRF47 |
| GQ175881 | CRF48 |
| GQ175882 | CRF48 |
| GQ175883 | CRF48 |
| HQ385477 | CRF49 |
| HQ385478 | CRF49 |
| HQ385479 | CRF49 |
| JN417236 | CRF50 |
| JN417240 | CRF50 |
| JN417241 | CRF50 |
| JN029801 | CRF51 |
| JN029803 | CRF51 |
| LC312713 | CRF51 |
| AY945734 | CRF52 |
| DQ354113 | CRF52 |
| DQ366664 | CRF52 |
| DQ366663 | CRF53 |
| JX390610 | CRF53 |
| JX390611 | CRF53 |
| EU031915 | CRF54 |
| JX390976 | CRF54 |
| JX390977 | CRF54 |
| JX574661 | CRF55 |
| JX574662 | CRF55 |
| JX574663 | CRF55 |
| JN882655 | CRF56 |
| KC852172 | CRF56 |
| KC852173 | CRF56 |
| KC852174 | CRF56 |
| HM776939 | CRF57 |
| JX679207 | CRF57 |
| KC870044 | CRF57 |
| KC522031 | CRF58 |
| KC522032 | CRF58 |
| KC522034 | CRF58 |
| JX960635 | CRF59 |
| KC462190 | CRF59 |
| KC462191 | CRF59 |
| KC899079 | CRF60 |
| KC899080 | CRF60 |
| KC899081 | CRF60 |
| KC990124 | CRF61 |
| KC990125 | CRF61 |
| KC990126 | CRF61 |
| KC870034 | CRF62 |
| KC870035 | CRF62 |
| KC870037 | CRF62 |
| JN230353 | CRF63 |
| JX500701 | CRF63 |
| JX500705 | CRF63 |
| KC870032 | CRF64 |
| KC870036 | CRF64 |
| KC870042 | CRF64 |
| KC870027 | CRF65 |
| KC870028 | CRF65 |
| KC870030 | CRF65 |
| KC183779 | CRF67 |
| KC183780 | CRF67 |
| KC183782 | CRF68 |
| KC183783 | CRF68 |
| KF758551 | CRF68 |
| AB845344 | CRF69 |
| AB845349 | CRF69 |
| LC027100 | CRF69 |
| KJ849758 | CRF70 |
| KJ849761 | CRF70 |
| KJ849809 | CRF70 |
| KU749388 | CRF70 |
| DQ358811 | CRF71 |
| KJ849759 | CRF71 |
| KJ849779 | CRF71 |
| KJ671534 | CRF72 |
| KJ671535 | CRF72 |
| KJ671536 | CRF72 |
| AY882421 | CRF73 |
| KM248765 | CRF73 |
| KR019770 | CRF74 |
| KR019771 | CRF74 |
| KR019772 | CRF74 |
| KX673818 | CRF77 |
| KX673819 | CRF77 |
| KX673820 | CRF77 |
| KU161143 | CRF78 |
| KU161144 | CRF78 |
| KU161145 | CRF78 |
| KY216146 | CRF79 |
| KY216147 | CRF79 |
| KY216148 | CRF79 |
| KU820825 | CRF82 |
| KU820831 | CRF82 |
| KU820836 | CRF82 |
| KU820845 | CRF82 |
| KU820834 | CRF83 |
| KU820842 | CRF83 |
| KU820843 | CRF83 |
| KU820847 | CRF83 |
| KU992928 | CRF85 |
| KU992929 | CRF85 |
| KU992930 | CRF85 |
| KX582249 | CRF86 |
| KX582250 | CRF86 |
| KX582251 | CRF86 |
| KC898992 | CRF87 |
| KC899012 | CRF87 |
| KF250408 | CRF87 |
| KC898975 | CRF88 |
| KC898979 | CRF88 |
| KF250402 | CRF88 |
| KY628218 | CRF90 |
| KY628221 | CRF90 |
| KY628223 | CRF90 |
| MF372645 | CRF92 |
| MF372647 | CRF92 |
| MF372648 | CRF92 |
| MF372650 | CRF92 |
| MF372646 | CRF93 |
| MF372649 | CRF93 |
| MF372651 | CRF93 |
| MG518476 | CRF96 |
| MG518477 | CRF96 |
| AF484509 | A1 |
| AB253429 | A1 |
| AF004885 | A1 |
| AF069670 | A1 |
| M62320 | A1 |
| AF286237 | A2 |
| AF286238 | A2 |
| AY521629 | A3 |
| AY521630 | A3 |
| AY521631 | A3 |
| AM000055 | A4 |
| AM000053 | A4 |
| AM000054 | A4 |
| AY331295 | B |
| AY423387 | B |
| K03455 | B |
| U63632 | B |
| M17451 | B |
| AY173951 | B |
| U21135 | B |
| U52953 | C |
| AF067155 | C |
| AF110967 | C |
| U46016 | C |
| AY772699 | C |
| AY371157 | D |
| U88822 | D |
| U88824 | D |
| K03454 | D |
| M27323 | D |
| AY253311 | D |
| AF005494 | F1 |
| AF075703 | F1 |
| AJ249238 | F1 |
| AF077336 | F1 |
| AY371158 | F2 |
| AF377956 | F2 |
| AJ249236 | F2 |
| AJ249237 | F2 |
| U88826 | G |
| AF084936 | G |
| AF061641 | G |
| AF061642 | G |
| AF005496 | H |
| AF190127 | H |
| AF190128 | H |
| AF082395 | J |
| AF082394 | J |
| AJ249235 | K |
| AJ249239 | K |
| AF286236 | L |
| AF457101 | L |
| AY046058 | U |

Supplemental Figure 1.

Supplemental Figure 1. The total number of samples sequenced (N) for each indicated virus(es) are plotted for each of the study sites.
